# Astrocyte-Specific Expression Patterns Associated with the PDGF-Induced Glioma Microenvironment

**DOI:** 10.1371/journal.pone.0032453

**Published:** 2012-02-29

**Authors:** Amanda M. Katz, Nduka M. Amankulor, Ken Pitter, Karim Helmy, Massimo Squatrito, Eric C. Holland

**Affiliations:** 1 Biochemistry, Cell, and Molecular Biology Program, Weill Medical College of Cornell University, New York, New York, United States of America; 2 Department of Cancer Biology and Genetics, Memorial Sloan-Kettering Cancer Center, New York, New York, United States of America; 3 Brain Tumor Center, Memorial Sloan-Kettering Cancer Center, New York, New York, United States of America; 4 Departments of Neurosurgery, Neurology and Surgery, Memorial Sloan-Kettering Cancer Center, New York, New York, United States of America; Dana-Farber Cancer Institute, United States of America

## Abstract

**Background:**

The tumor microenvironment contains normal, non-neoplastic cells that may contribute to tumor growth and maintenance. Within PDGF-driven murine gliomas, tumor-associated astrocytes (TAAs) are a large component of the tumor microenvironment. The function of non-neoplastic astrocytes in the glioma microenvironment has not been fully elucidated; moreover, the differences between these astrocytes and normal astrocytes are unknown. We therefore sought to identify genes and pathways that are increased in TAAs relative to normal astrocytes and also to determine whether expression of these genes correlates with glioma behavior.

**Methodology/Principal Findings:**

We compared the gene expression profiles of TAAs to normal astrocytes and found the Antigen Presentation Pathway to be significantly increased in TAAs. We then identified a gene signature for glioblastoma (GBM) TAAs and validated the expression of some of those genes within the tumor. We also show that TAAs are derived from the non-tumor, stromal environment, in contrast to the Olig2+ tumor cells that constitute the neoplastic elements in our model. Finally, we validate this GBM TAA signature in patients and show that a TAA-derived gene signature predicts survival specifically in the human proneural subtype of glioma.

**Conclusions/Significance:**

Our data identifies unique gene expression patterns between populations of TAAs and suggests potential roles for stromal astrocytes within the glioma microenvironment. We show that certain stromal astrocytes in the tumor microenvironment express a GBM-specific gene signature and that the majority of these stromal astrocyte genes can predict survival in the human disease.

## Introduction

Gliomas are the most common primary malignant brain tumor in adults [1]. Current treatment options for glioma include surgical resection, radiation therapy and chemotherapy. Unfortunately, even with these aggressive treatments, patient response is poor and average survival of patients with aggressive forms of glioma is less than 2 years [2]. One possible reason for poor patient response to treatment may be that standard glioma therapy regimen do not account for the effects of stromal cells within the tumor microenvironment [3]. There is a lack of stroma-directed therapy due to the paucity of data clarifying the role of local non-transformed cells in glioma maintenance and progression. As the role of the tumor microenvironment becomes increasingly relevant it is important to identify stromal cells and factors that may contribute to tumor growth.

Recent advances in characterizing gene expression profiles from patient samples have divided gliomas into four main subgroups (Classical, Mesenchymal, Neural and Proneural) each highlighted by the activation or loss of specific signaling pathways [4]–[6]. Notable genetic alterations in high-grade gliomas include loss of NF1, increased EGFR signaling and increased PDGF signaling [5]. The Proneural subgroup of gliomas, which is characterized by increased PDGF signaling [5], [7], can be effectively modeled using the RCAS/tv-a system of retroviral gene transfer of PDGF into Nestin-expressing cells in neonatal mouse brains [8], [9]. This genetically engineered tumor model recapitulates the biology of human gliomas in an immunocompetent host, allowing for the study of transformed cells as well as stromal cells in the tumor microenvironment. In this PDGF-induced model of glioma, tumors of varying grades arise and can be characterized in a histologically accurate manner. Low-grade gliomas exhibit proliferation of tumor cells surrounded by a relatively quiescent stromal architecture (WHO grade II), whereas high-grade tumors are histologically similar to WHO grade III and IV and feature proliferating cells in addition to marked microvascular proliferation (Grades III and IV) and pseudopalisading necrosis (Grade IV). In the PDGF-driven model of glioma, the tumor bulk is comprised of oligodendrocyte-type cells. However the tumor microenvironment contains many cell types that may contribute to tumor growth and maintenance. Among these cells, astrocytes are relatively abundant in the tumor microenvironment and may contribute to tumor growth. While the histology of TAAs has been described [10], the biological role of these astrocytes is largely unexplored.

Astrocytes are often identified by their expression of Glial Fibrillary Acidic Protein (GFAP), which increases in response to injury. Astrocytes were originally thought to play a passive role in the brain, but they are now known to play critical roles in supporting the integrity of the blood brain barrier [11] and the transmission of neural impulses [12]. In addition, astrocytes contribute to the brain's response to injury. When injury occurs, astrocytes become reactive, change their morphology, proliferate and migrate to the area of injury. Depending on the context of injury, they may promote and/or inhibit neurogenesis [13], [14]. Recently, our group found that reactive astrocytes can induce proliferation of Olig2-expressing cells after injury [15]. This finding raises the intriguing possibility that astrocytes may be associated with expanding oligodendrocyte cell populations in other central nervous system disease states, including neoplasms.

In addition to reactive astrocytes, adult neural stem cells (aNSC) also express GFAP [16]. These cells share ultrastructural and protein expression characteristics with mature astrocytes [17] and reside in two neurogenic niches within the adult brain: the sub-ventricular zone of the lateral ventricles and the sub-granular zone in the dentate gyrus of the hippocampus [18]. Within these regions, the neural stem cells reside adjacent to blood vessels [19], [20] and these adult astrocyte-like stem cells proliferate slowly and are thought to divide asymmetrically [21] to give rise to progenitor cells that proliferate rapidly and symmetrically to produce terminal cell types such as neurons [22]–[24] and glia [23], [25].

Recent studies have highlighted the plasticity of astrocytes with regard to their functions in injury and as stem cells. In addition to shared markers and ultrastructural characteristics between reactive astrocytes and aNSC, recent work has demonstrated that reactive astrocytes derived from an injury setting possess characteristics specific to aNSC, including the expression of aNSC markers as well as the ability to proliferate and generate neurospheres [11], [14], [26], [27]. These studies suggest a novel role for astrocytes in the context of injury and demonstrate the plasticity of reactive astrocytes.

The role of astrocytes can vary significantly among gliomas. In PDGF-driven gliomas where the majority of transformed cells closely resemble oligodendrocytes, astrocytes appear to play a supportive role in the perivascular stem cell niche and the peri-tumoral (invasive) edge of these tumors. In contrast, the bulk of the tumor cells in astrocytomas are astrocytic in morphology. The biology of transformed astrocytes derived from astrocytomas, including the genetic alterations and gene expression patterns in these cells, has been studied in depth. However, little is known about the TAAs found in PDGF-driven oligodendrogliomas. Considering the growth-promoting and stem cell properties of astrocytic cells in normal physiologic states and brain injuries and the possibility that these properties can be co-opted by the tumor to promote proliferation and progression, we were compelled to study the gene-expression properties of TAAs in PDGF-driven gliomas with the goal of identifying stromal factors that contribute to tumorigenesis.

To study TAAs, we used a GFAP-GFP transgenic mouse that expresses GFP under the control of the human GFAP promoter, which allows for isolation of astrocytes using fluorescence activated cell sorting (FACS) from normal and tumor brains. When compared to normal astrocytes, samples enriched for TAAs show markedly increased expression of members of the antigen presentation pathway. Additionally, we identified genes specific to TAAs in GBM and validate their expression. We also demonstrate that TAAs are derived from the stromal microenvironment and that many of the gene expression changes seen in TAAs are specifically present in stroma and not in tumor cells. Further, we demonstrate that many of these differentially expressed genes predict survival in the human proneural subtype of glioma. These data suggest a role for stromal astrocyte-expressed genes in tumor progression and identifies potential therapeutic targets in the treatment of proneural gliomas.

## Results

### Tumor-associated Astrocytes are a Component of the Glioma Microenvironment

To generate tumors that mimic the proneural subtype of glioma, we injected the RCAS-PDGF avian retrovirus into *Ink4a/Arf* heterozygous mice that express the RCAS receptor tv-a under the control of the Nestin promoter. Using this model, *Ink4a/Arf* heterozygous mice injected with RCAS-PDGF develop tumors of varying grades within four to eight weeks. Upon manifestation of brain tumor symptoms such as weight loss and poor grooming, mice were sacrificed and their brains were fixed and embedded in paraffin for histologic analysis and grading. Using RCAS-PDGF in the *Ink4a/Arf* heterozygous background allows for the generation of tumors of varying grades within the same litter (Figure S1A).

**Figure 1:**
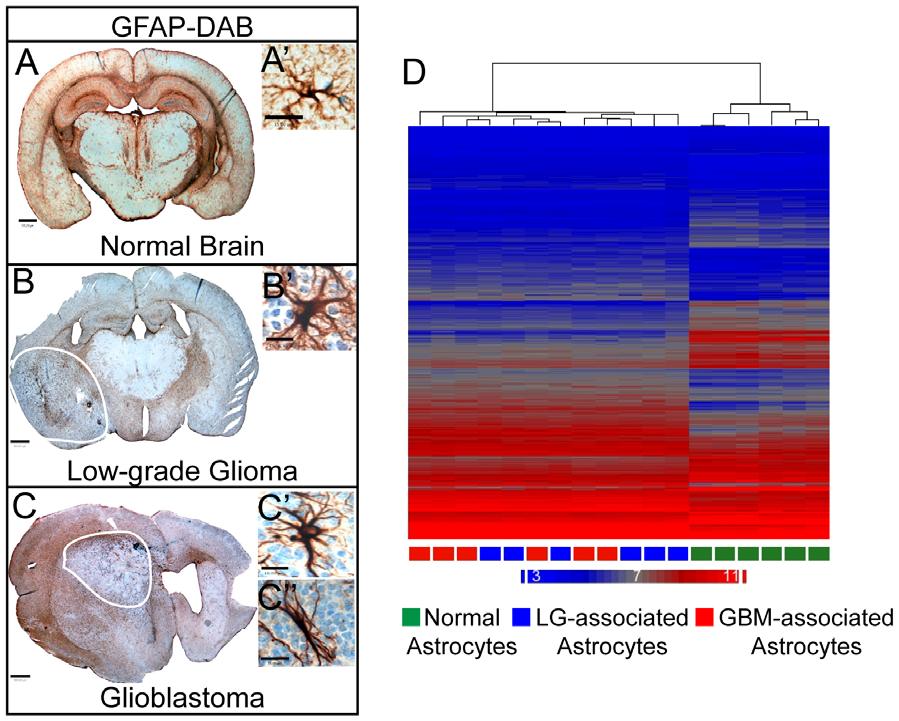
Tumor-associated Astrocytes within PDGF-driven Glioma. (A–C) GFAP immunohistochemistry of astrocytes in the normal brain (A, A′), WHO II low-grade glioma (B, B′) and glioblastoma (GBM; C, C′, C″) at 1× (A, B, C) and 40× (A′, B′, C′, C″). Note that tumor-associated astrocytes (TAAs) are morphologically different than normal astrocytes. Moreover, in low grade glioma, TAAs are present within and surrounding the tumor and all of these astrocytes have a ‘reactive’ morphology identified by swollen cell bodies as well as multipolar and hyperextended processes (B′). Within GBM (C), astrocytes are present in two areas: the peri-tumoral area, where the astrocytes have a ‘reactive’ morphology (C′) similar to low grade astrocytes and the perivascular niche, where the astrocytes still have swollen cell bodies but have a more uni-polar or bi-polar morphology (C″). Scale bars: A, B, C = 300 µm, A′, B′, C′, C″ = 15 µm. D) Unbiased hierarchical clustering of astrocytes from normal brain, low-grade glioma and GBM indicates that, when factoring in the mRNA expression levels of approximately 15,000 genes significantly expressed on the array, TAAs are very different from normal astrocytes, however most genes are similarly regulated between low grade-associated and GBM-associated astrocytes and thus, low grade-associated and GBM-associated astrocytes do not segregate.

As described above, the tumor bulk in the PDGF-driven model of glioma expresses Olig2, while the GFAP-expressing cells are part of the tumor stroma. These stromal astrocytes are geographically and morphologically different from normal astrocytes (Figure 1A). In low-grade gliomas, the astrocytes are present throughout the tumor in a diffuse pattern (Figure 1B). These astrocytes are morphologically similar to reactive astrocytes, with swollen cell bodies and processes extending in multiple directions (Figure 1B). In the PDGF-induced GBMs, astrocytes are present in the tumor in two distinct areas: the peri-tumoral region, located at the tumor periphery, and the perivascular niche which is hypothesized to harbor the stem cell niche in glioblastoma [28] (Figure 1C). The peri-tumoral astrocytes have swollen cell bodies and processes that extend out in multiple directions (Figure 1B′, 1C′). This population of astrocytes surrounds the tumor in a manner similar to the way in which reactive astrocytes surround an area of injury [11] and this population of astrocytes is present in tumors of all grades (Figure 1B,1C). The perivascular astrocytes, however, are only present at a significant level in high-grade glioma, where significant microvascular proliferation is present [29]. We also noted that these astrocytes have a more bipolar morphology [24] (Figure 1C″) and are localized to areas surrounding the tumor blood vessels (Figure 1C, Figure S1B) [19], [20]. The perivascular astrocytes also express the stem cell marker Nestin [10].

### The mRNA Profile of Tumor-associated Astrocytes Differs from Normal Astrocytes

In order to determine the differences between normal astrocytes and TAAs and to identify genes and pathways increased in TAAs we generated PDGF-driven gliomas in GFAP-GFP transgenic mice, which express GFP under the control of the human GFAP promoter [30], and used FACS to specifically collect samples enriched for TAAs from microdissected low-grade gliomas and GBM. We used FACS and histology to confirm that the GFP-positive cells represented TAAs and not immune or endothelial cells (Figure S2A–D). Normal astrocytes were collected from 6 week-old mice that were not injected with the RCAS virus. Collected astrocytes were resuspended in trizol, their RNA was extracted and cDNA was run on illumina-6 arrays and Partek software was used to analyze samples. Unbiased hierarchical clustering analysis of all genes significantly represented on the array showed that TAAs exhibit similar expression patterns regardless of tumor grade, indicating a shared phenotype, and that TAAs differ markedly from normal astrocytes (Figure 1D).

### MHC Class II Pathway Is Active In Tumor-associated Astrocytes

In order to identify genes and pathways significantly changed in TAAs, we generated a list of genes that were increased or decreased by four-fold in both low-grade glioma and GBM TAAs relative to normal astrocytes. We specifically characterized low-grade tumors as WHO grade II tumors (lacking vascular proliferation) and GBM as tumors exhibiting both hypervascularity and pseudo-pallisading necrosis (WHO grade IV). We then used ingenuity pathway analysis (IPA) to query pathways associated with the resulting genes and identified the antigen presentation pathway as the most significantly represented pathway increased in TAAs relative to normal astrocytes (Table 1). While previously published data has shown astrocytes to have antigen-presenting capabilities *in vitro* as well as in *in vivo* injury models [31]–[35], this function of stromal astrocytes has not been identified in a glioma mouse model. We used immunoflourescent staining to confirm MHC class II expression on TAAs in low-grade gliomas and GBM (Figure S3A) and stained with Iba-1, an immune cell marker, to demonstrate that these MHC class II-expressing astrocytes were not microglia or macrophages. We also used FACS analysis to demonstrate an increase in MHCII expression on TAAs when compared to normal astrocytes (Figure S3B,C). In addition to MHC class II, our array analysis identified other components of antigen presentation pathway as being increased in TAAs (Table S1).

**Table 1:** Antigen Presentation Pathway is Active in Tumor-associated Astrocytes.

Ingenuity Canonical Pathways	-log(p-value)	Ratio
Antigen Presentation Pathway	1.08E+01	2.56E-01
Dendritic Cell Maturation	7.81E+00	8.05E-02
Type I Diabetes Mellitus Signaling	6.05E+00	8.70E-02
Crosstalk between Dendritic Cells and Natural Killer Cells	5.55E+00	9.18E-02
Virus Entry via Endocytic Pathways	4.65E+00	8.33E-02
Caveolar-mediated Endocytosis Signaling	4.30E+00	8.43E-02
B Cell Development	4.00E+00	1.32E-01
CXCR4 Signaling	3.70E+00	5.39E-02
Allograft Rejection Signaling	3.69E+00	1.11E-01
Graft-versus-Host Disease Signaling	3.69E+00	1.11E-01

© 2000–2010 Ingenuity Systems, Inc. All rights reserved.

Ingenuity Pathway Analysis of genes significantly increased more than four-fold in low-grade glioma- and glioblastoma-associated astrocytes indicates the pathways that are significantly increased in TAAs relative to normal astrocytes. The top ten canonical pathways represented on the array are shown in the table. The Antigen Presentation Pathway is most represented within this gene set.

### Comparative Expression Identifies a Stromal Gene Signature for GBM Tumor-associated Astrocytes

**Figure 2:**
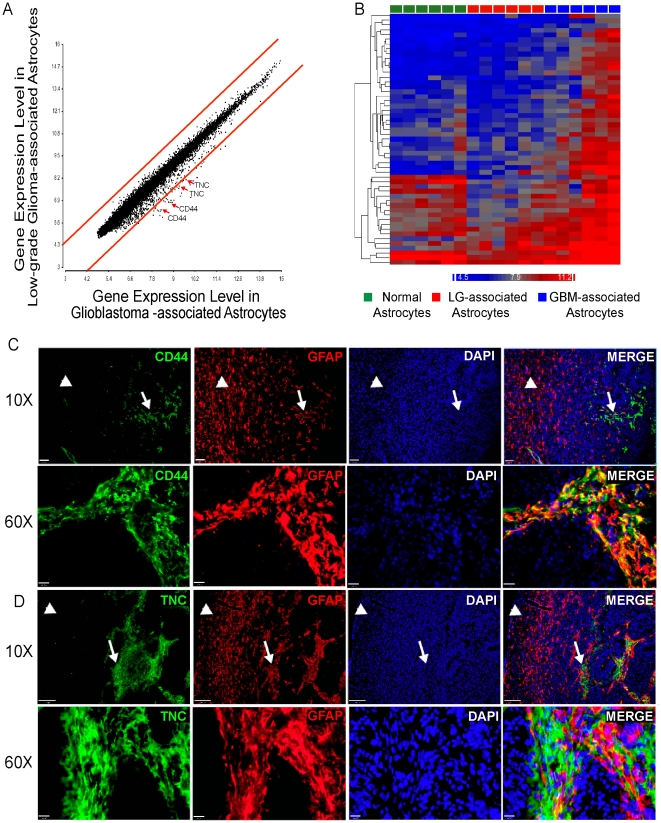
A Gene Signature for GBM Astrocytes. A) Dot-plot of the expression levels of all genes significantly expressed in low-grade-associated astrocytes and GBM-associated astrocytes. Red lines demarcate genes similarly expressed regardless of grade and dots outside the red lines represent genes expressed at higher levels in GBM-associated astrocytes. Red arrows point to the dots representing *Cd44* and *Tenascin C.* B) Hierarchical clustering of the genes off the plot that are expressed at a higher level in GBM-associated astrocytes relative to low-grade-associated astrocytes indicates that these genes are able to segregate GBM-associated astrocytes from low grade-associated astrocytes. C,D) Validation of CD44 (C) and Tenascin-C (TNC; D) expression at low- and high-magnification. CD44 and TNC are expressed at high levels in perivascular astrocytes but expressed at lower levels or not expressed at all in peri-tumoral astrocytes. Arrows point to perivascular astrocytes and arrowheads point to peri-tumoral astrocytes. Scale bars = 100 µm, 10 µm.

In order to identify stromal genes important for tumorigenesis, we next looked specifically for genes expressed at much higher levels in GBM TAAs when compared to TAAs from low-grade gliomas. Having established that the astrocytes from low-grade glioma and GBM were relatively similar, we hypothesized that there may be a subset of genes differentially expressed between the two populations and that these genes may play a role in tumor progression. In order to identify genes specific to GBM TAAs we plotted gene expression data in TAAs from low-grade gliomas and compared it to that of TAAs from GBMs (Figure 2A). Since the perivascular niche is only present in GBM [29], high-grade gliomas would be expected to have a more robust representation of perivascular astrocytes and thus some differentially expressed genes in GBM TAAs may represent genes specific to the perivascular astrocytes. Our data show that the mRNA expression profiles in TAAs are relatively similar regardless of grade (Figure 1D, Figure 2A), however, a small subset of GBM-specific, astrocyte-associated genes emerged from this analysis (Figure 2A, Table 2). In fact, using this gene signature in hierarchical clustering analysis enabled us to differentiate GBM gene expression from low-grade glioma (Figure 2B). We screened a number of antibodies to these GBM-specific genes using immunofluorescence and chose two (Tenascin C and CD44) for further analysis. CD44 and Tenascin C show clear expression in astrocytes (Figure 2C,D). Moreover, their expression is virtually restricted to perivascular astrocytes. We also noted that CD44, a known stem cell marker [36], [37], is only expressed in specific astrocytes within the perivascular niche (Figure S4A) as well as in GFAP-expressing neurospheres (Figure S4B). However, CD44 does not stain astrocytes that reside further from the ventricle (Figure S4A) or cultured, differentiated astrocytes (Figure S4B,C).

**Table 2:** A Small Group of Genes are Expressed Only in GBM-associated Astrocytes.

Gene Symbol	Fold-Change in GBM-associated vs. Low grade-associated Astrocytes
Spp1	7.38667
Mmp10	5.59439
Serpina3h	4.74814
Col6a1	4.74803
Cd44	4.52953
Ctgf	4.33288
Col6a3	4.32895
Vgf	4.28414
Ctgf	4.27893
Lyz	4.23631
S100a11	4.13884
Mglap	4.13362
Aldh1a3	4.03689
Timp1	3.95154
Cd44	3.90811
Timp1	3.89863
Lyzs	3.86976
Emp1	3.82806
Col6a1	3.69735
Lcn2	3.57219
Igfbp7	3.47077
Sphk1	3.45682
Prss19	3.36531
Nupr1	3.31251
Col18a1	3.27824
C1qb	3.27781
Lgals3	3.2697
Anxa2	3.15955
Lyzs	3.10612
Hk2	3.06335
Arhgdib	3.00618
Tnc	2.99419
9130006A14Rik	2.98302
Tnc	2.97213
Ppic	2.89373
Ccl4	2.8926
Bgn	2.88815
C1qg	2.88698
Aldh1a3	2.85672
9230117N10Rik	2.85186
0610041G09Rik	2.83913
Lgals1	2.8309
Rbp1	2.81274
Anxa3	2.787
Col18a1	2.75882
Dlk1	2.71719
Cd109	2.70004
Cdkn1a	2.69406

A list of the genes expressed at higher levels in GBM-associated astrocytes when compared to low-grade-associated astrocytes. Genes are in order of the difference of expression between GBM-associated and low grade-associated-astrocytes. CD44 and TNC are each represented twice on the list. Osteopontin, which is at the top of the list, is a ligand for the CD44 receptor.

### Tumor-associated Astrocytes Are Frequently Derived From the Stromal Environment

**Figure 3:**
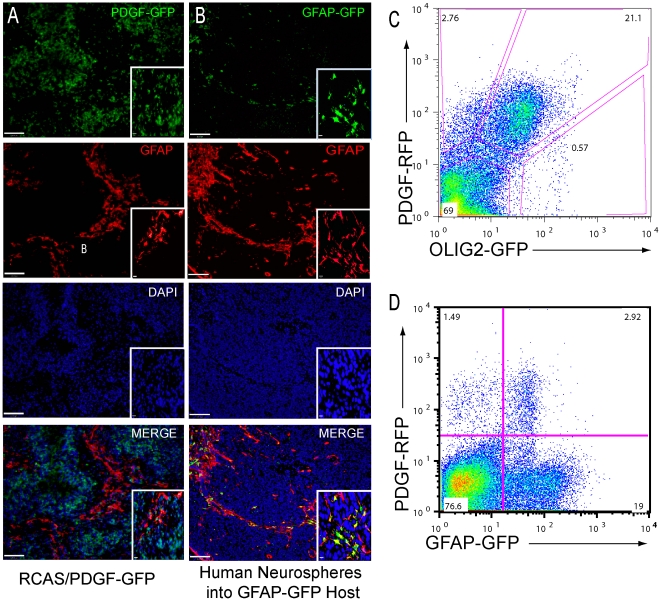
Perivascular Astrocytes Can Be Stromal in Origin. A) Immunofluorescence for PDGF-GFP (green) and GFAP (red) in a PDGF-GFP-driven glioma shows expression of PDGF-GFP and GFAP are mutually exclusive, indicating that TAAs are not derived from the tumor cell of origin. Scale bars = 100 µm. Inset, high-magnification images. Scale bars = 10 µm. B) Immunofluorescence for GFAP (red) and GFP (green) in an orthotopic model of human glioma. Human tumorspheres were injected into GFAP-GFP reporter mice such that tumor derived astrocytes will express GFAP but not GFP and host/stromal astrocytes will co-express GFAP and GFP. While some astrocytes express only GFAP, many astrocytes also express GFP and are thus derived from the host stromal environment. Expression of stromal GFAP-GFP occurs in all areas of GFAP immunoreactivity. Scale bars = 25 µm. Inset, high-magnification images. Scale bars = 10 µm. C) FACS analysis of a PDGF-RFP-driven glioma in an Olig2-rp-GFP mouse, where PDGF-infected tumor cells express RFP and Olig2-expressing cells express GFP. Most cells infected with the PDGF-RFP virus are Olig2-rp-GFP-expressing cells. D) FACS analysis of a PDGF-RFP-driven glioma in a GFAP-GFP mouse, where PDGF-infected cells express RFP and astrocytes express GFP. Most of the TAAs were not infected with RCAS-PDGF-RFP.

Because astrocytes are an abundant component of the tumor microenvironment, we asked whether TAAs were derived from the RCAS-transduced tumor cell of origin or from the stromal microenvironment. To achieve this goal, we utilized fluorescently labeled RCAS-PDGF vectors combined with immunofluorescence and FACS analysis. We generated gliomas using RCAS-PDGF-GFP [10], [38]. We then used GFP immunofluorescence to identify tumor progeny cells and stained the same tissue for GFAP to identify astrocytes and look for co-localization. In these PDGF-driven gliomas, PDGF-GFP infected tumor cells and GFAP-expressing astrocytes were expressed in mutually exclusive areas (Figure 3A), confirming that the astrocytes in our tumors are stromal. We validated this data using a model of human orthotopic glioma injected into GFAP-GFP host mice. In this orthotopic model, TAAs derived from tumor cells would not express the GFAP-GFP transgene; conversely, TAAs derived from non-tumor cells should express GFP. When we injected non-fluorescent human tumorspheres into GFAP-GFP host mice, we noted robust correlation of GFP and GFAP expression, consistent with the hypothesis that these TAAs are host derived. (Figure 3B). While the orthotopic tumors exhibited a smaller degree of microvascular proliferation than mouse tumors, all perivascular areas within the tumors contained host-derived, GFP+, GFAP-expressing cells. Importantly, this finding implies that host derived TAAs are present in the tumor stem cell niche.

Since GFAP-expressing cells have extensive processes with indistinct boundaries, immunostaining may not always be the ideal way to differentiate host-derived astrocytes from astrocytes that are derived from injected tumors. We therefore used FACS analysis to determine whether GFAP-expressing cells were derived from tumor cells or the non-neoplastic microenvironment. For these experiments we used an RCAS-PDGF-mRFP virus to generate tumors in GFAP-GFP [30] and Olig2-rpGFP mice [39]. Analysis of tumors generated in Olig2-rpGFP mice infected with RCAS-PDGF-RFP showed that most of the PDGF-RFP infected cells were Olig2-expressing cells, consistent with the idea of an oligodendrocyte lineage in tumor cells derived by PDGF over-expression (Figure 3C). Correspondingly, tumors generated by injecting RCAS-PDGF-mRFP into GFAP-GFP transgenic mice, showed little to no expression of PDGF-RFP within the GFAP-GFP population (Figure 3D). It is worth noting that a large percentage of PDGF-RFP infected cells expressed low levels of GFP and, given our staining and FACS data, these represent PDGF-infected Olig2+ tumor cells that also express low levels of GFAP [40] (Figure 3A, C). Finally, as a first step toward exploring the biological properties of these stromal TAAs, we assessed their ability to generate neurospheres *in vitro* and found that they generated neurospheres under the appropriate conditions (Figure S4D). This property is reminiscent of injury-associated reactive astrocytes, which also display neurosphere-generating capacity [14].

### Expression Levels of GBM-associated Astrocytic Stromal Genes Predict Survival in Human Proneural GBMs Specifically

**Figure 4:**
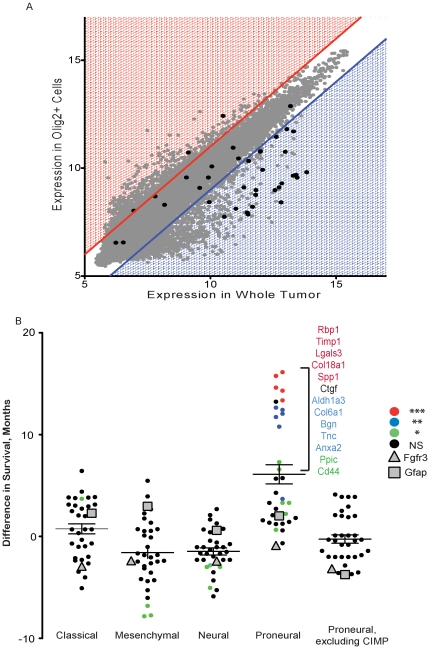
GBM Astrocyte-associated Stromal Genes Can Predict Survival in Human Proneural Glioma. A) Dot-plot of the expression levels of all genes significantly expressed in whole tumor extracts (tumor plus stroma) versus Olig2-expressing tumor cells only. Red shaded area demarcates genes expressed at a higher level in Olig2-expressing tumor cells, whereas blue shaded area demarcates genes expressed at a higher level in Olig2-negative, stromal cells. Black dots represent genes identified as GBM TAAs signature genes. B) Analysis of the predictive value of the GBM TAA genes. For each gene, patients were divided into high expressers and low expressers, based on whether their expression of the gene was above or below the median. Kaplan Meier curves were then generated for the two groups and the difference in survival between low and high expressers was plotted. Black dots indicate a non-significant difference in survival and colored dots indicate a significant difference in survival, where red is *** (p<0.001), blue is ** (p<0.01), and green is * (p<0.05).

We next sought additional confirmation that GBM TAAs genes were expressed in the stromal compartment of the tumor microenvironment and not in the tumor cells. To do this, we plotted the gene expression data from whole tumors relative to the gene expression data from the Olig2-expressing tumor cells that had been sorted from the rest of the tumor. This analysis enables us to identify genes specific to stromal cells, as these genes would only be expressed in the whole tumor population and not in the purified Olig2+ tumor cell population. Using this analysis, most of the genes identified as specific to GBM TAAs when compared to low grade (WHO Grade II) TAAs were also identified as arising only in the stromal compartment of the tumor and not in the Olig2-expressing tumor cells (Figure 4A). In fact, of the 38 genes identified, only 3 genes (8%) were expressed at higher levels in the tumor cells, whereas 24 genes (63%) were expressed at significantly higher levels in whole tumor relative to Olig2 tumors cells and 11 genes (29%) did not demonstrate a significant expression difference between Olig2 cells and whole tumors. Taken together, these data confirm that genes expressed in GBM TAAs are expressed predominately in the stromal microenvironment rather than in the Olig2-expressing tumor cells.

Once we confirmed the stromal nature of the GBM TAAs signature genes, we were interested in knowing whether these stromal genes play a role in tumorigenesis. Moreover, given the differences in the astrocytic component of proneural gliomas compared to non-proneural tumors, we were interested in exploring the possibility that proneural TAAs play a role in the molecular pathophysiology of proneural glioma. In order to understand whether our GBM TAA signature is active in human tumors, we turned to the TCGA dataset [6] and asked whether expression of these genes can predict survival in any given tumor subtype. For each gene, patients were divided into two groups, low expressing and high expressing, based on the median expression of a given gene within a given subtype. For all four subtypes of glioma, we determined the average survival of patients based on their expression levels of each gene and plotted the difference in survival between low and high expressing patients (Figure 4B, Figure S5). In the proneural subtype, 17 of the 38 genes (45%) showed a significant, inverse correlation with survival, where high expression was associated with worse prognosis. Further, of these 17 genes, 12 of them are associated with a significant survival difference of more than 6 months and of those, 10 genes predict a significant survival difference of more than 1 year. Additionally, 8 of the 12 genes that predict a difference in survival of more than 6 months are enriched in the stromal compartment. Notably, most of these genes did not significantly correlate with survival in the Classical, Mesenchymal or Neural subtypes of glioma. In addition, neither GFAP nor FGF3, two established astrocyte markers [11], [41] are associated with survival, indicating that these correlations are specific to gene expression, rather than a simple indication of astrocyte density present in the tumor microenvironment. These data indicate that stromal astrocyte gene expression patterns within the proneural subtype of glioma correlate with patient outcome and may serve as useful biomarkers for the proneural subtype of glioma.

Recent data has identified the presence of a CpG island methylator phenotype (CIMP) within the Proneural subgroup [42]. These tumors have hypermethylated promoter CpG islands, which results in transcriptional silencing of the associated genes. The glioma-CIMP (G-CIMP) tumors have a more favorable prognosis when compared to non-CIMP Proneural tumors or gliomas within the Classical, Mesenchymal and Neural subtypes. We therefore explored whether the CIMP phenotype altered the prognostic potential of our GBM TAAs signature genes. Remarkably, we found that the GBM TAAs signature genes can only predict survival in the Proneural subtype when the G-CIMP samples are included, and not when the G-CIMP samples are excluded from the data set (Figure 4B, Figure S5). In fact, while expression of these genes is low in the Proneural subtype in general, it is even lower in the G-CIMP tumors (Figure S6), indicating a connection between these genes or stromal astrocytes and G-CIMP tumors.

## Discussion

Our understanding of astrocyte function in neurological disease has increased vastly in recent years. Whereas astrocytes were once thought to be post-mitotic, negative regulators of tissue growth, we now know that astrocytes possess proliferative and progenitor cell functions [14], [15]. The majority of the data on astrocyte function comes from injury models, neurodegenerative disorders, transformed astrocytes in glial tumors, and the study of a small subset of astrocyte-like adult neural stem cells. In this study, we identify some basic characteristics of stromal astrocytes within the PDGF-driven tumor microenvironment. Furthermore, we identify TAA markers that are associated with survival in the human proneural subtype of glioma.

Astrocytes respond to injury by changing their morphology, proliferating and migrating to the site of injury [11]. Here we show that in our PDGF-driven model of proneural gliomas, astrocytes respond to tumors in a manner reminiscent of their response in brain injury. TAAs surrounding the tumor are morphologically ‘reactive’ with swollen cell bodies, hyperextended processes extending in multiple directions, and they express high levels of GFAP. In an injury model, these astrocytes are capable of inducing proliferation of Olig2-expressing cells [15] and it is possible that this pro-proliferative astrocyte function may be directed to Olig2-expressing tumor cells in proneural gliomas.

TAAs are not only structurally similar to injury-response astrocytes but are also similar on a molecular level. Specifically, they are thought to express Sonic Hedgehog [10], [15] as well as members of the antigen presentation pathway. The antigen presentation pathway is mainly comprised of MHC class I and class II molecules. These molecules are important for antigen presentation to T cells. While previously published data has shown that astrocytes possess antigen-presenting capabilities *ex vivo* and in models of brain injury [31]–[35], this function of astrocytes has not been identified in a glioma mouse model and highlights a novel and potentially important role for TAAs.

In addition to the antigen presentation pathway, we also identified a gene signature that distinguishes GBM-associated astrocytes from those in lower grade gliomas. These genes are mainly expressed in the stromal compartment of the tumor, and are associated with survival in the proneural subtype of human glioma. Two of these markers, CD44 and Tenascin C, which are expressed in the stem cell niches of normal brains [37], [43]–[45], are expressed at much higher levels in the perivascular niche where tumor stem cells are proposed to reside [28]. Additionally, the fact that CD44 and Tenascin C are expressed at much higher levels in the perivascular astrocytes when compared to peritumoral astrocytes suggests subtle differences between the two populations of non-neoplastic tumor astrocytes. We will direct some of our future studies towards understanding whether these gene expression changes produces functional differences in tumor pathophysiology.

To further understand characteristics of peritumoral and perivascular TAAs, we investigated whether these cells were derived from transformed cells, stroma, or both cellular compartments. We expected to find that peritumoral astrocytes were stromal in origin, but were intrigued to learn that perivascular astrocytes found in the center of these tumors were also stromal in origin. While it is tempting to attribute functional roles to these astrocytes that exist at the core of the tumor stem cell niche, more investigation is necessary before such conclusion can be reached.

It is not currently known where the stromal-derived perivascular astrocytes originate. Numerous studies have shown that normal neural stem cells demonstrate a tropism towards tumors [46], [47] and the migration of normal neural stem cells out of the SVZ and SGZ and into the tumor perivascular niche is a possibility. Another explanation is that these perivascular astrocytes are simply local astrocytes that become “trapped” within the perivascular niche. A corollary to the latter hypothesis is that the presence of secreted PDGF and robust microvasculature causes these astrocytes to assume a more stem/progenitor phenotype and dedifferentiate. This hypothesis is supported by previous work has shown that reactive astrocytes are capable of stem-like behavior [14].

In addition to identifying stromal genes expressed in TAAs of the PDGF-driven mouse gliomas, we identified that a subset of these stromal genes are prognostic of survival in the human proneural GBMs, where high expression was associated with worse outcome. In fact, almost half of the genes identified were able to predict a difference in survival of at least 1 year. Moreover, the predictive value of these genes is specific to the proneural subtype of glioma, indicating the possibility of a TAA effect within the proneural subtype of human glioma. Interestingly, the predictive value of these genes was lost when the g-CIMP patients were removed. Three of the stromal genes (Rbp1, Lgals3 and Anxa2) were methylated at a significantly higher level in g-CIMP tumors compared to non-CIMP tumors [42], and even unmethylated genes were significantly downregulated in g-CIMP tumors when compared to non-CIMP tumors [42]. The association of these genes with the g-CIMP tumors suggests the possibility of an altered tumor microenvironment in the g-CIMP proneural tumors when compared to the non-CIMP tumors. Further, that these genes were identified within the stromal compartment raises the question of the effects of the g-CIMP tumor cells on the non-neoplastic stromal cells within the tumor.

Our data highlight the importance of the tumor microenvironment, specifically TAAs, in the proneural subtype of glioma. These TAAs respond to the tumor in the microenvironment and express genes that are associated with overall patient survival. As more is learned about the various pathways driving the neoplastic growth of gliomas, we must also consider the role of the stromal cells within these tumors. If these cells are capable of promoting tumor growth, they may need to be targeted in treatment protocols. Because these cells are not neoplastic, conventional mutagenic treatments may not be effective and thus, the ability to target specific growth-promoting pathways in these cells is imperative. It will be important to identify other genes and pathways active in stromal cells, but the identification of the antigen presentation pathway as well as GBM TAAs signature genes provides us with a starting point in elucidating the functional roles of tumor microenvironment in progression and malignancy.

## Methods

### Generation of brain tumors

Nestin-tv-a (*N-tva*) transgenic mice were injected with DF1 cells (purchased from ATCC) producing RCAS-PDGFB [8], RCAS-PDGF-sv40-GFP [10] or RCAS-PDGF-RFP [48] as previously described [10]. Mice were monitored carefully for symptoms of tumor development (lethargy, hydrocephalus, head tilting). GFAP-GFP mice were purchased from Jackson Laboratories and crossed onto the *Ntv-a, Ink4a/Arf* null background to generate tumors that were heterozygous for *Ink4a/Arf* and GFAP-GFP. Olig2-rp-GFP [38], [39] mice were also crossed onto the *Ntv-a, Ink4a/Arf* null background to generate tumors that were heterozygous for *Ink4a/Arf* and Olig2-rp-GFP. For human orthotopic tumors, 100,000 cells from human tumorsphere cultures [49] were injected into host mice which were then monitored carefully for symptoms of tumor development and sacrificed upon manifestation of these symptoms. All animal work was conducted using protocols approved by the Institutional Animal Care and Use Committee at Memorial Sloan-Kettering Cancer Center. The approved protocols are 00-11-189 (MSKCC, last approved 3/15/2010).

### Cell Culture

DF1 cells overexpressing RCAS-PDGF, RCAS-PDGF-sv40-GFP or RCAS-PDGF-mRFP were maintained in media containing 10% FBS in a humidified atmosphere containing 5% carbon dioxide at 39 degrees. Neurosphere cultures were maintained in basal media (Stem Cell Technologies) supplemented with 5% proliferation supplement (Stem Cell Technologies), EGF and FGF, as previously described [50].

### Immunohistochemistry and Immunofluorescence

For paraffin embedded samples, tumors were fixed in 10% formalin and embedded in paraffin. Histological grading was determined based on Hematoxylin and Eosin staining and the presence of high-grade structures. DAB staining was performed using the Ventana DAB-MAP system. The following antibodies were used for DAB staining: GFAP (1∶10,000, DAKO), CD44 (1∶1000, BD Biosciences). Immunofluorescence was performed on 10% formalin fixed, paraffin embedded samples as well as on 4% paraformaldehyde fixed tissues embedded in OCT and frozen. For paraffin embedded tissue: tissues were deparaffinized and rehydrated in graded alcohols. Antigen unmasking was performed at 93° for 15 minutes. Tissues were then permeabilized in 0.3% triton-x/PBS, blocked in 5% serum and stained overnight in primary antibody. The next morning, samples were washed in 0.1% triton-X/PBS and stained in the appropriate alexa-fluor tagged secondary antibody for 2 hours at room temperature. Samples were then counterstained with DAPI and mounted in 70% glycerol or prolong gold (Invitrogen). For frozen samples: tissues were air-dried and then permeabilized, blocked and stained as described above. The following antibodies were used for fluorescent staining: GFAP (1∶2000, Dako), CD44 (1∶1000, BD Pharmingen), MHCII (212-A1 antibody, 1∶1000, a gift from Dr, Lisa Denzin), IBA-1 (1∶500 Wako), Nestin (1∶100, BD Pharmingen) Tenascin C (1∶100, Abcam).

### FACS Sorting/Analysis

For analysis and sorting of tumors, tumors were enzymatically digested in EBSS containing 12% papain (Worthington) and 10 mg/ml DNase at 37°C for 15 minutes. The digestion was stopped with 1 mg/ml ovomucoid (Worthington). Cells were washed and resuspended in basal medium and sorted using a Cytomation MoFlo cell sorter. Cells to be used on the array were collected in PBS, centrifuged and resuspended in trizol. Cells that were to be cultured were collected directly into the appropriate media. For analysis and sorting of primary neurospheres, spheres were mechanically dissociated, resuspended in basal media and sorted using a Beckton-Dickinson FACSCalibur flow cytometer. Antibodies used for FACS include CD44-PE (1∶100, BD Pharmingen), CD44-Pacific blue (1∶100, Biolegend), APC-conjugated MHC-II (1∶500, ebiosciences), CD45-APC (1∶100, BD Pharmingen), CD31-APC (1∶100, BD Pharmingen).

### Array

GFAP-GFP-expressing astrocytes were collected by FACS from *Ink4a/Arf* heterozygous mice with or without PDGF-driven gliomas. Prior to sorting, a piece of the tumor was removed for histological analysis of grade. Six samples each of Normal, low-grade-associated and GBM-associated astrocytes were collected, pelleted and then resuspended in trizol. Three samples of Olig2-rpGFP cells and three samples of whole tumors were resuspended in trizol. RNA was extracted from normal astrocytes as well as from samples enriched for TAAs and Illumina-6 arrays were run by the genomics core at MSKCC. For the Olig2-rpGFP versus whole tumor array, Illumina-8 arrays were used. Array data was analyzed using Partek analysis software. Data was imported as a text file and was quantile and then log2 normalized prior to analysis. ANOVA statistics were run to determine significantly changed genes. Gene lists were uploaded into Ingenuity Pathway Analysis for analysis of relevant pathways.

### Analysis of Human Data

Expression values for each gene of interest were obtained from the MSKCC computational biology cancer genomics portal, http://www.cbioportal.org/cgx/index.do, which has annotated TCGA data. Patient survival data was obtained from published TCGA information [6]. For each subtype of tumors, the mean and median expression for each GBM TAAs gene was determined. Patients were then divided into two groups (‘high’ and ‘low’), depending on whether their expression of the gene was above or below the median, and Kaplan Meier curves were generated based on the resulting data. The difference in survival between patients with high expression of a given gene compared to patients with low expression of that gene was then calculated for each gene in each subtype and that difference in survival was plotted on a new graph.

### Statistical Analysis

Array statistics were performed using ANOVA in the Partek analysis software. Comparison of the changes in MHC-II expression in astrocytes and CD44 expression in differentiated and neurosphere conditions, as well as the differences in gene expression between subtypes was performed using a two-tailed t-test in prism software.

## Supporting Information

Figure S1 **Astrocytes in Glioma.** A) Examples of Low-grade Gliomas and GBMs at low- and high-magnification. B) Immunofluorescence of tumors from Lectin-GFP injected mice stained with GFAP (red) demonstrates that astrocytes within the tumor reside close to blood vessels. Scale bars = 10 µm.
(TIF)

Figure S2 ***Gfap*** **-GFP Reporter Recapitulates Endogenous GFAP Expression.** A) Immunofluorescence for GFAP (red) and GFP (green) in the hippocampus of a normal brain from a GFAP-GFP mouse shows colocalization of the GFP reporter with GFAP protein. Scale bars = 50 µm. B) Immunofluorescence for GFAP (red) and GFP (green) in a tumor-bearing brain shows colocalization of the GFP reporter with GFAP protein. Scale bars = 10 µm C) FACS analysis indicates that GFAP-GFP cells do not colocalize with CD31-expressing endothelial cells. GFAP-GFP expression is on the x-axis; CD31 expression is on the y-axis. D) FACS analysis indicates that GFAP-GFP cells do not colocalize with CD45-expressing immune cells. GFAP-GFP expression is on the x-axis; CD45 expression is on the y-axis.
(TIF)

Figure S3 **MHC Class II Expression is Increased in Tumor-associated Astrocytes When Compared to Normal Astrocytes.** A) Immunofluorescence for MHC II (red), GFAP (green) and Iba-1 (white) in low grade- and GBM-associated astrocytes shows co-localization of MHC II with GFAP-expressing astrocytes. Arrowheads point to MHC II-expressing astrocytes that are Iba-1 negative and thus not immune cells. Scale bars = 10 µm B) FACS analysis of normal and tumor-bearing brains for expression of MHC class II (y-axis) and GFAP-GFP (x-axis). In normal brains, there is very little MHC II expression and virtually no expression on astrocytes. However, in a tumor-bearing brain, MHCII expression is increased in the tumor and specifically within the astrocyte population. C) Quantification of results of FACS analysis: in a normal brain, approximately 1.5% of astrocytes express MHCII and in a tumor brain, approximately 17.5% of astrocytes express MHCII, p = 0.0351.
(TIF)

Figure S4 **CD44 Expression in Astrocytes.** A) Immunofluorescent staining for CD44 (red) and GFAP (green) in the sub-ventricular zone of a normal brain. Arrows point to CD44-expressing astrocytes near the ventricle (V). In addition to expressing CD44, these astrocytes have a bipolar morphology, which is a hallmark of adult astrocyte-like stem cells [24]. Arrowheads point to CD44-negative astrocytes further from the ventricle. In addition to being far from the ventricle, these astrocytes have processes extending in multiple directions, indicating that these cells are not adult astrocyte-like stem cells [24]. Scale bars = 10 µm. B) FACS analysis of tumors cultured in serum-containing media or neurosphere media demonstrates that CD44 expression is only maintained in neurosphere conditions. Also, when normal astrocytes are cultured as neurospheres they express CD44. CD44 expression is on the x-axis and GFAP-GFP expression is on the y-axis. C) Quantification of FACS data. After one week, approximately 50% of TAAs cultured in stem-like conditions express CD44. D) Host-derived, murine GFAP-GFP-expressing astrocytes from an orthotopic model of human glioma were sorted, collected and grown as neurospheres. Within these cultures there is significant expression of GFAP-GFP, indicating the ability of stromal astrocytes to grow as neurospheres. Scale bars = 100 µm.
(TIF)

Figure S5 **GBM TAAs Genes can Predict Survival in Human Proneural Glioma.** Representative Kaplan Meier curves for the GBM TAAs genes in each subtype as well as G-CIMP only tumors. Blue line represents survival of patients with expression below the median for each gene in each subtype and black line represent survival for patients with expression above the median for each gene in each subtype, * = p<0.05, ** = p<0.01, *** = p<0.001. Survival is represented in months to death after diagnosis.
(TIF)

Figure S6 **G-CIMP Patients Have Lower Expression of GBM TAAs Genes.** Plot of average expression of the GBM TAAs signature genes in each subtype and in proneural G-CIMP and non-G-CIMP patients. The average expression for all GBM TAAs genes was lowest in the proneural subtype and lower in proneural G-CIMP patients when compared to proneural non-G-CIMP patients. * = p<0.05, ** = p<0.01, *** = p<0.001.
(TIF)

Table S1 **MHC Class II Pathway is increased in Tumor-associated Astrocytes Relative to Normal Astrocytes.** List of MHC Class II pathway genes that are increased in TAAs compared to normal astrocytes. Values listed as fold change in expression when TAAs were compared to normal astrocytes.
(TXT)
