# Supplementary figures and images for: Astrocyte-Specific Expression Patterns Associated with the PDGF-Induced Glioma Microenvironment
Source: PLoS One. 2012 Feb 29;7(2):e32453. doi: 10.1371/journal.pone.0032453 (PMC3290579; doi:10.1371/journal.pone.0032453)

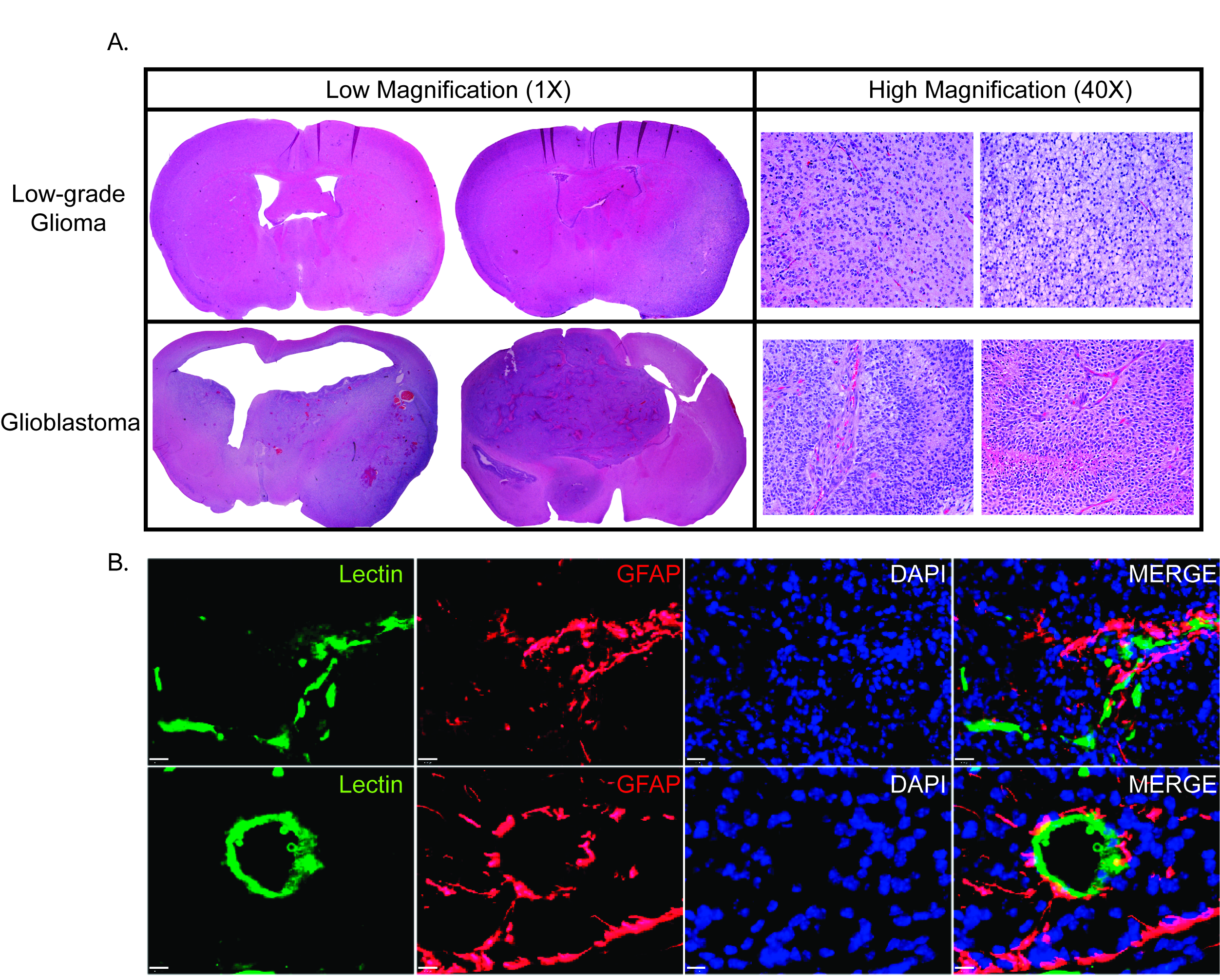

Supplement: Figure S1 — Astrocytes in Glioma. A) Examples of Low-grade Gliomas and GBMs at low- and high-magnification. B) Immunofluorescence of tumors from Lectin-GFP injected mice stained with GFAP (red) demonstrates that astrocytes within the tumor reside close to blood vessels. Scale bars = 10 µm. (TIF) [file pone.0032453.s001.tif]

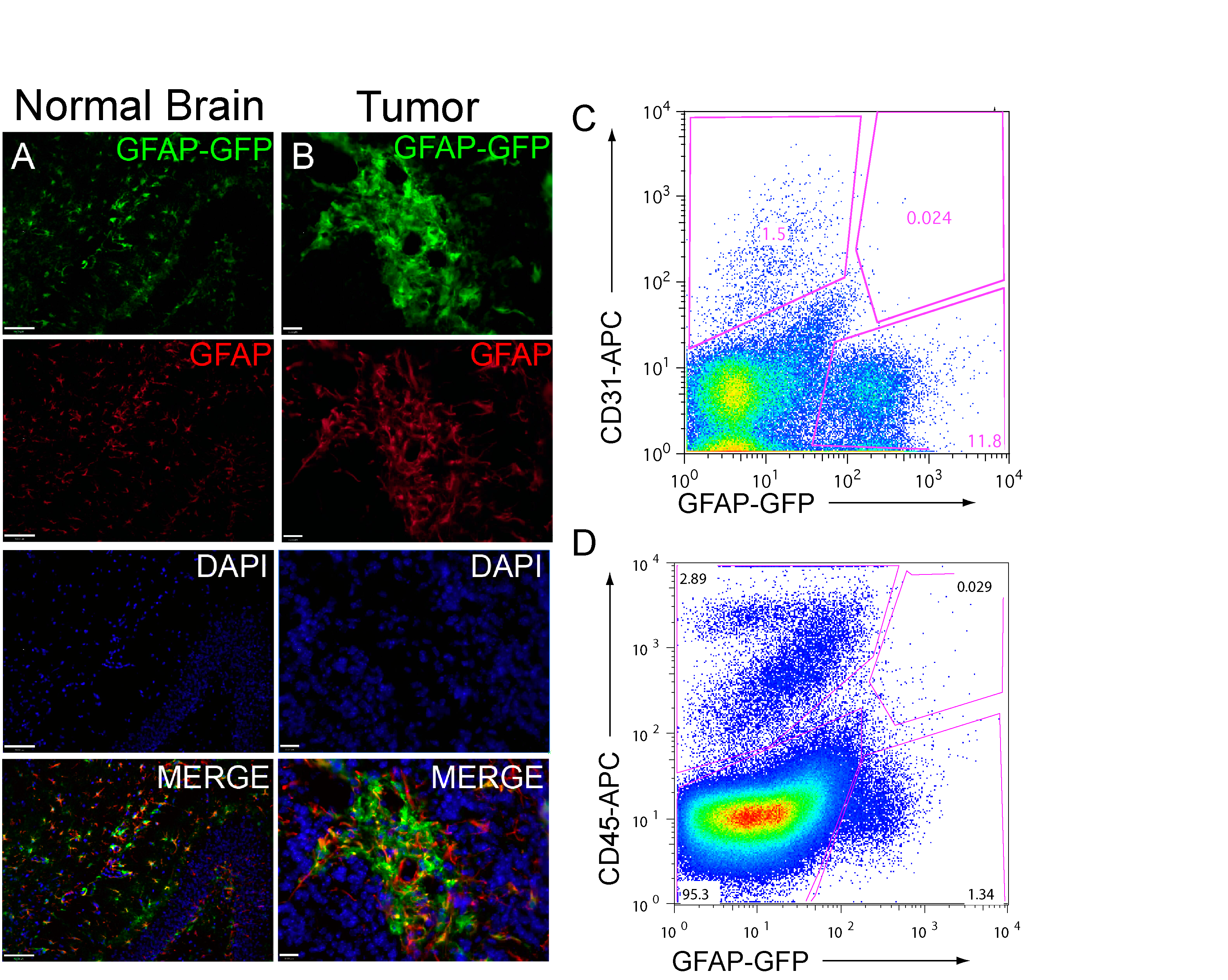

Supplement: Figure S2 — Gfap -GFP Reporter Recapitulates Endogenous GFAP Expression. A) Immunofluorescence for GFAP (red) and GFP (green) in the hippocampus of a normal brain from a GFAP-GFP mouse shows colocalization of the GFP reporter with GFAP protein. Scale bars = 50 µm. B) Immunofluorescence for GFAP (red) and GFP (green) in a tumor-bearing brain shows colocalization of the GFP reporter with GFAP protein. Scale bars = 10 µm C) FACS analysis indicates that GFAP-GFP cells do not colocalize with CD31-expressing endothelial cells. GFAP-GFP expression is on the x-axis; CD31 expression is on the y-axis. D) FACS analysis indicates that GFAP-GFP cells do not colocalize with CD45-expressing immune cells. GFAP-GFP expression is on the x-axis; CD45 expression is on the y-axis. (TIF) [file pone.0032453.s002.tif]

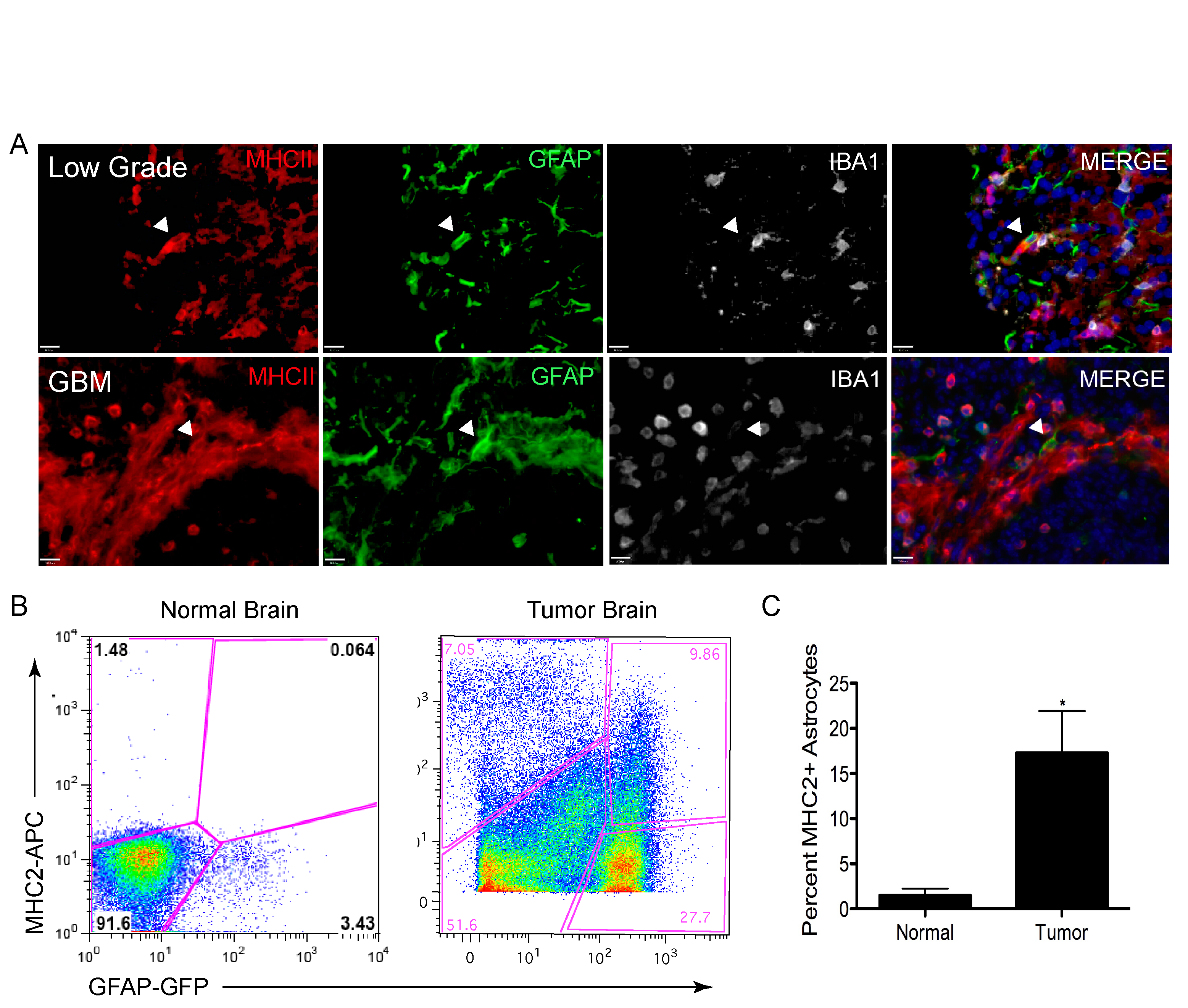

Supplement: Figure S3 — MHC Class II Expression is Increased in Tumor-associated Astrocytes When Compared to Normal Astrocytes. A) Immunofluorescence for MHC II (red), GFAP (green) and Iba-1 (white) in low grade- and GBM-associated astrocytes shows co-localization of MHC II with GFAP-expressing astrocytes. Arrowheads point to MHC II-expressing astrocytes that are Iba-1 negative and thus not immune cells. Scale bars = 10 µm B) FACS analysis of normal and tumor-bearing brains for expression of MHC class II (y-axis) and GFAP-GFP (x-axis). In normal brains, there is very little MHC II expression and virtually no expression on astrocytes. However, in a tumor-bearing brain, MHCII expression is increased in the tumor and specifically within the astrocyte population. C) Quantification of results of FACS analysis: in a normal brain, approximately 1.5% of astrocytes express MHCII and in a tumor brain, approximately 17.5% of astrocytes express MHCII, p = 0.0351. (TIF) [file pone.0032453.s003.tif]

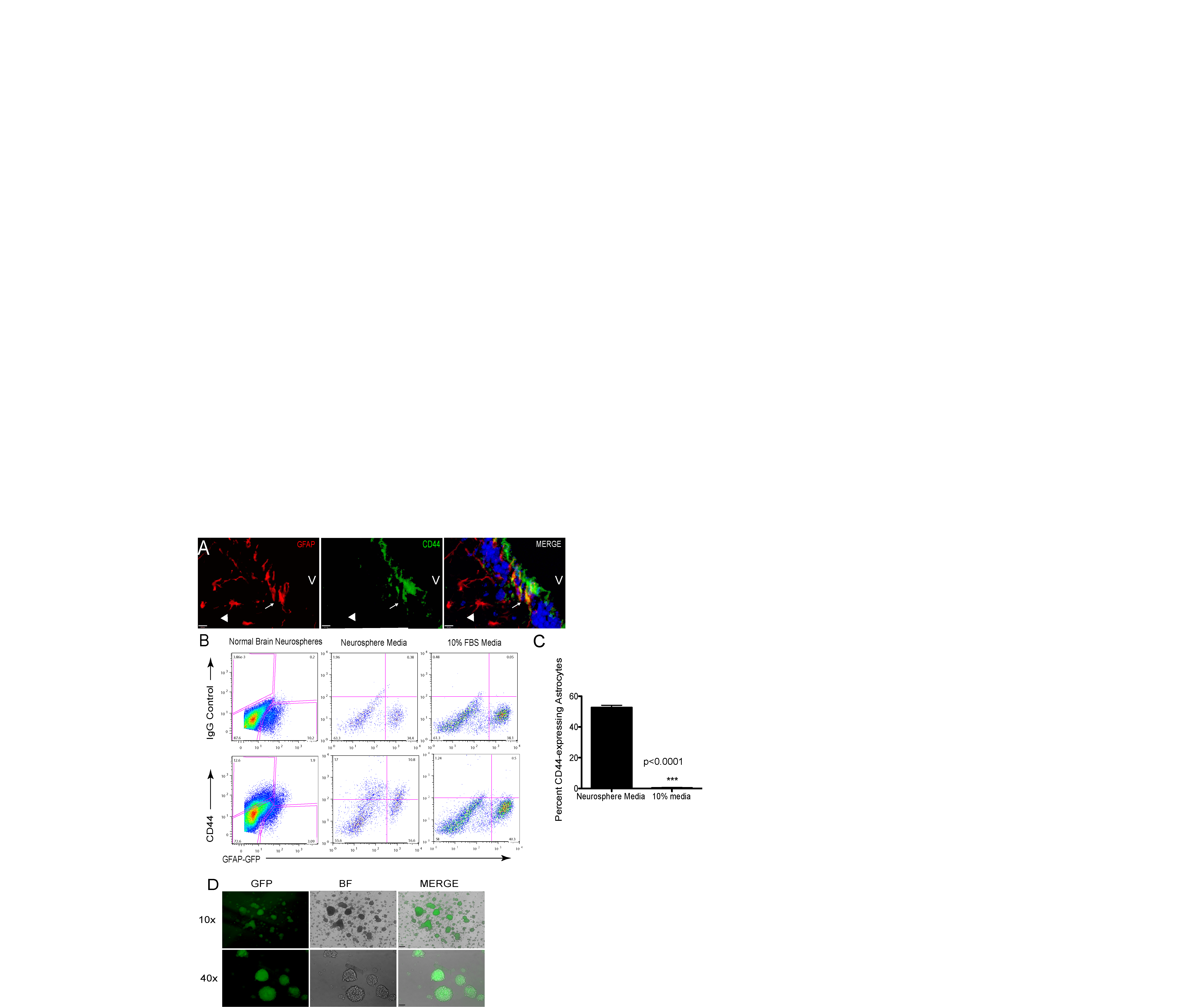

Supplement: Figure S4 — CD44 Expression in Astrocytes. A) Immunofluorescent staining for CD44 (red) and GFAP (green) in the sub-ventricular zone of a normal brain. Arrows point to CD44-expressing astrocytes near the ventricle (V). In addition to expressing CD44, these astrocytes have a bipolar morphology, which is a hallmark of adult astrocyte-like stem cells [24]. Arrowheads point to CD44-negative astrocytes further from the ventricle. In addition to being far from the ventricle, these astrocytes have processes extending in multiple directions, indicating that these cells are not adult astrocyte-like stem cells [24]. Scale bars = 10 µm. B) FACS analysis of tumors cultured in serum-containing media or neurosphere media demonstrates that CD44 expression is only maintained in neurosphere conditions. Also, when normal astrocytes are cultured as neurospheres they express CD44. CD44 expression is on the x-axis and GFAP-GFP expression is on the y-axis. C) Quantification of FACS data. After one week, approximately 50% of TAAs cultured in stem-like conditions express CD44. D) Host-derived, murine GFAP-GFP-expressing astrocytes from an orthotopic model of human glioma were sorted, collected and grown as neurospheres. Within these cultures there is significant expression of GFAP-GFP, indicating the ability of stromal astrocytes to grow as neurospheres. Scale bars = 100 µm. (TIF) [file pone.0032453.s004.tif]

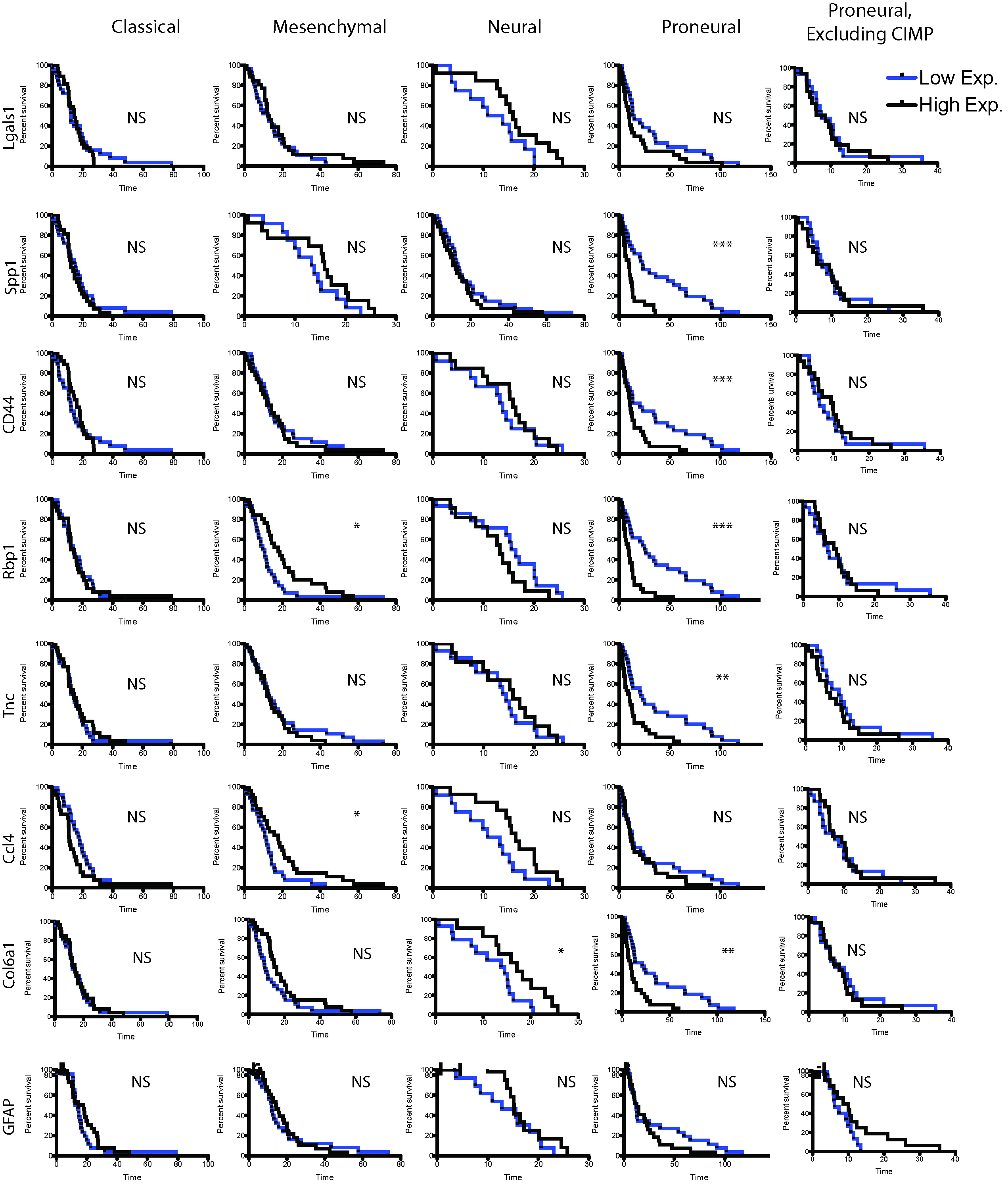

Supplement: Figure S5 — GBM TAAs Genes can Predict Survival in Human Proneural Glioma. Representative Kaplan Meier curves for the GBM TAAs genes in each subtype as well as G-CIMP only tumors. Blue line represents survival of patients with expression below the median for each gene in each subtype and black line represent survival for patients with expression above the median for each gene in each subtype, * = p<0.05, ** = p<0.01, *** = p<0.001. Survival is represented in months to death after diagnosis. (TIF) [file pone.0032453.s005.tif]

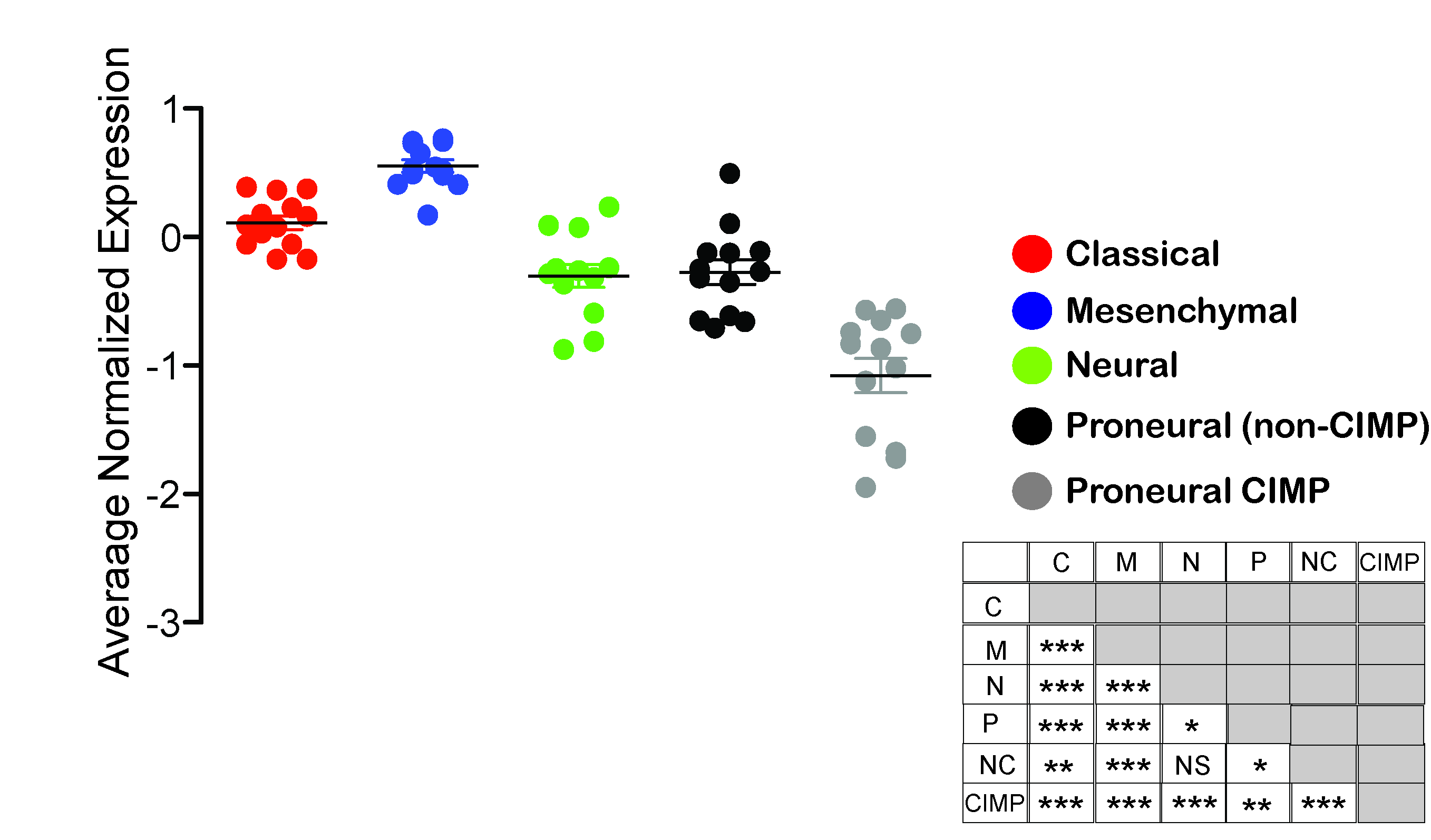

Supplement: Figure S6 — G-CIMP Patients Have Lower Expression of GBM TAAs Genes. Plot of average expression of the GBM TAAs signature genes in each subtype and in proneural G-CIMP and non-G-CIMP patients. The average expression for all GBM TAAs genes was lowest in the proneural subtype and lower in proneural G-CIMP patients when compared to proneural non-G-CIMP patients. * = p<0.05, ** = p<0.01, *** = p<0.001. (TIF) [file pone.0032453.s006.tif]
